# Supplementary figures and images for: Urbanicity, biological stress system functioning and mental health in adolescents
Source: PLoS One. 2020 Mar 18;15(3):e0228659. doi: 10.1371/journal.pone.0228659 (PMC7080241; doi:10.1371/journal.pone.0228659)

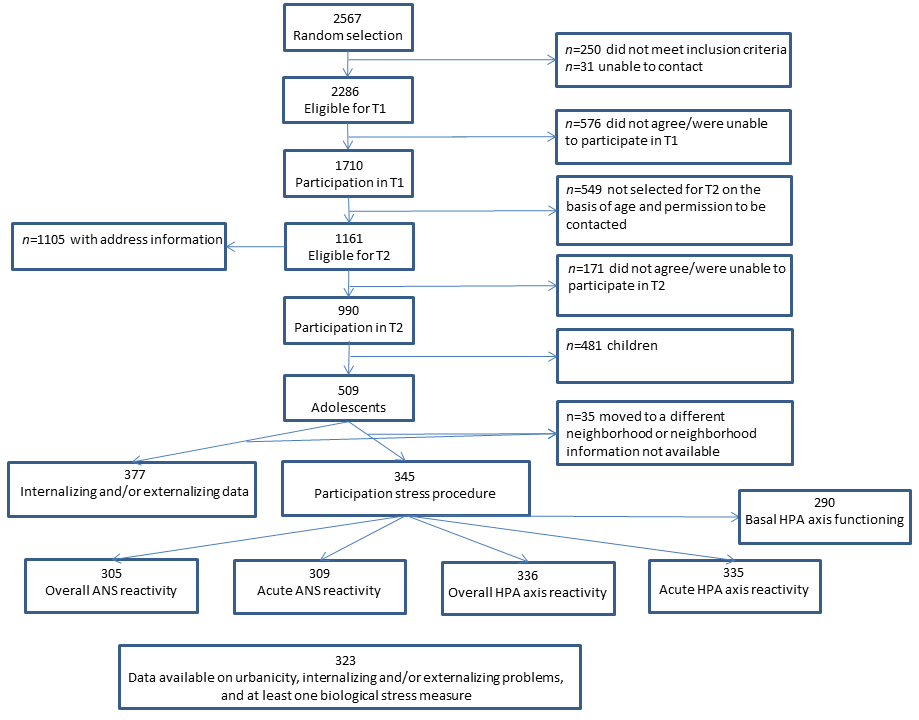

Supplement: S1 Fig — (TIF) [file pone.0228659.s001.tif]

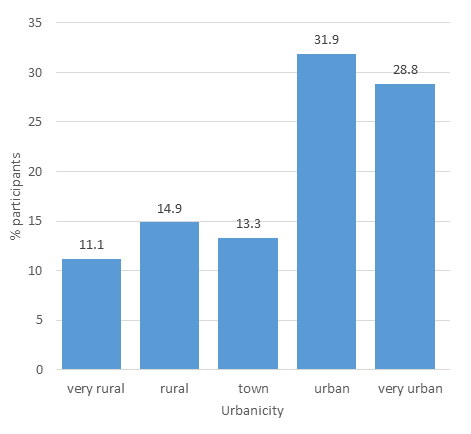

Supplement: S2 Fig — The urbanicity score is calculated using the surrounding address density (SAD) and coded as very rural (average SAD < 500 addresses per km2), rural (average SAD between 500 and 1000 addresses per km2), town (average SAD between 1000 and 15000 addresses per km2), urban (average SAD between 1500 and 2500 addresses per km2), and very urban (average SAD ≥ 2500 addresses per km2). In the analyses, the urbanicity scale was utilized as a continuous measure. (TIF) [file pone.0228659.s002.tif]
