# Supplementary material for: Urbanicity, biological stress system functioning and mental health in adolescents
Source: PLoS One. 2020 Mar 18;15(3):e0228659. doi: 10.1371/journal.pone.0228659 (PMC7080241; doi:10.1371/journal.pone.0228659)
Supplement: S1 File — Participants were shown a feelings thermometer (available at https://www.pearsonclinical.nl/adis-c-complete-set) as they were asked these questions. Translation was for the purpose of providing supplementary information and was not done systematically. (DOCX) [file pone.0228659.s003.docx]

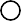

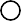

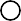

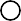
GesteldheidsVragenlijst 1 2 3 4 5


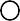


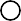


**(Perceived Stress Questionnaire)**

We willen je vragen om op de thermometer steeds aan te geven in hoeverre je de volgende dingen voelt:

(We would like to ask you to show us on the thermometer how much you feel the following things)

1. Heb je warme of plakkerige handen?

(Are your hands warm or sticky?)

1. Ben je aan het zweten?

(Are you sweating?)

1. Voel je je hart kloppen?

(Can you feel your heart beating?)

1. Heb je het heel erg warm of zelfs benauwd?

(Are you hot or do you feel weak?)

1. Heb je een droge mond?

(Does your mouth feel dry?)

1. Voel je je vingers of gezicht tintelen

(Do you feel your fingers or your face tingling?)

1. Ben je zenuwachtig?

(Are you nervous?)

Observatie proefleider: (Test leader’s observations)

.............................................

.............................................

**.............................................**

.............................................

.............................................

.............................................

SCORE:

SCORE:

SCORE:

SCORE:

SCORE:

SCORE: SCORE:
