# Supplementary material for: Urbanicity, biological stress system functioning and mental health in adolescents
Source: PLoS One. 2020 Mar 18;15(3):e0228659. doi: 10.1371/journal.pone.0228659 (PMC7080241; doi:10.1371/journal.pone.0228659)
Supplement: S1 Table — AUCi = area under the curve with respect to increase; MR = maximum response; AUCg = area under the curve with respect to ground; SES = socioeconomic status; l/a/h = low/average/high. For continuous variables (i.e. behavioral problems, emotional problems, urbanicity, all biological stress variables, neighborhood SES variables and age) t and d statistics are reported, for categorical variables (i.e. family SES and sex) χ2 and φ statistics are reported. (DOCX) [file pone.0228659.s004.docx]

S1 Table

|  | **In final models** | | **Not in final models** | | **Sample differences** | | |
| --- | --- | --- | --- | --- | --- | --- | --- |
|  | N | M(SD)/F(%) | N | M (SD)/F(%) | *t* or χ^2^ | *p* | *d* or *φ* |
| Behavioral problems: self | 298 | 1.40(0.73) | 39 | 1.19(0.59) | -2.04 | .05 | .32 |
| Emotional problems: self | 298 | 0.89(0.68) | 39 | 0.88(0.76) | -0.08 | .94 | .01 |
| Behavioral problems: mother | 304 | 0.78(0.71) | 47 | 0.85(0.80) | 0.56 | .58 | .09 |
| Emotional problems: mother | 304 | 0.57(0.58) | 48 | 0.60(0.53) | 0.28 | .78 | .05 |
| Urbanicity | 323 | 2.52(1.34) | 185 | 2.60(1.27) | 0.64 | .52 | .06 |
| Heart rate (AUCi) | 275 | 4639.88(609.83) | 30 | 4585.42(539.12) | -0.52 | .61 | .09 |
| Heart rate (MR) | 277 | 10.21(8.59) | 32 | 10.17(7.18) | -0.03 | .98 | .01 |
| Cortisol (AUCi) | 301 | 329.23(212.61) | 35 | 282.84(156.37) | -1.59 | .12 | .25 |
| Cortisol (MR) | 300 | 1.08(3.39) | 35 | 1.07(2.50) | -0.02 | .98 | .00 |
| Cortisol (AUCg) | 267 | 7154.37(2524.03) | 23 | 6740.90(2765.14) | -0.69 | .50 | .16 |
| SES Employment | 323 | 0.17(0.86) | 186 | -0.03(1.08) | -2.18 | .03 | .20 |
| SES Income | 323 | 0.05(0.95) | 186 | -0.24(0.82) | -3.55 | <.001 | .33 |
| Family SES (l/a/h) | 307 | 11/25/64 | 179 | 15/30/55 | 3.82 | .15 | .09 |
| Age | 323 | 17.07(1.51) | 186 | 16.77(1.58) | -2.11 | .04 | .19 |
| Sex (boy/girl) | 323 | 46/54 | 186 | 52/48 | 1.71 | .19 | .06 |
