# Supplementary material for: Urbanicity, biological stress system functioning and mental health in adolescents
Source: PLoS One. 2020 Mar 18;15(3):e0228659. doi: 10.1371/journal.pone.0228659 (PMC7080241; doi:10.1371/journal.pone.0228659)
Supplement: S2 Table — Intraclass correlations (ICC) were calculated using empty models. The ICC1 indicates the percentage of variance in behavioral and emotional problems that can be explained by group (i.e. neighborhood) membership. The ICC2 is an indication of reliability and should be > .70 [113]. AUCiHR = area under the curve with respect to ground, calculated for heart rate; MRHR = maximum heart rate response; AUCiC = area under the curve with respect to increase, calculated for cortisol; MRC = maximum cortisol response; AUCgC = area under the curve with respect to ground, calculated for cortisol. (DOCX) [file pone.0228659.s005.docx]

S2 Table

|  | ICC1 | ICC2 |
| --- | --- | --- |
| Behavioral problems |  |  |
| Mother-report | .00 | .00 |
| Adolescent-report | .03 | .03 |
| Emotional problems |  |  |
| Mother-report | .00 | .00 |
| Adolescent-report | .00 | .00 |
| AUCiHR | .00 | .00 |
| MRHR | .10 | .11 |
| AUCiC | .00 | .00 |
| MRC | .05 | .05 |
| AUCgC | .00 | .00 |
