# Supplementary material for: Urbanicity, biological stress system functioning and mental health in adolescents
Source: PLoS One. 2020 Mar 18;15(3):e0228659. doi: 10.1371/journal.pone.0228659 (PMC7080241; doi:10.1371/journal.pone.0228659)
Supplement: S3 Table — All statistics are Greenhouse-Geisser corrected. Main effects of time: perceived stress model df = 3.08, cortisol model df = 2.43, heart rate model df = 2.98. For all contrasts: df = 1. Pre-task cortisol value pertains to the lower value of RC1 and RC2. MAT = mental arithmetic task; PST = public speaking task; prep = preparation; CT = computer task. (DOCX) [file pone.0228659.s006.docx]

S3 Table

|  | **Perceived stress** | | | **Cortisol** | | | **Heart rate** | | |
| --- | --- | --- | --- | --- | --- | --- | --- | --- | --- |
|  | *F* | *p* | *η^2^* | *F* | *p* | *η^2^* | *F* | *p* | *η^2^* |
| *Main effect* |  |  |  |  |  |  |  |  |  |
| Time | 220.30 | < .001 | .42 | 65.85 | < .001 | .19 | 300.87 | < .001 | .53 |
| *Simple contrasts* |  |  |  |  |  |  |  |  |  |
| Pre-task *vs* MAT | 105.38 | < .001 | .26 | 2.91 | .09 | .01 | 195.39 | < .001 | .42 |
| Pre-task *vs* PST prep |  |  |  |  |  |  | 74.38 | < .001 | .22 |
| Pre-task *vs* PST speech | 181.63 | < .001 | .37 | 0.00 | .99 | .00 | 217.90 | < .001 | .45 |
| Pre-task *vs* CT | 0.45 | .50 | .00 | 28.54 | < .001 | .09 | 29.25 | < .001 | .10 |
| Post-task *vs* MAT |  |  |  | 158.25 | < .001 | .35 |  |  |  |
| Post-task *vs* PST speech |  |  |  | 157.32 | < .001 | .35 |  |  |  |
| Post-task *vs* CT |  |  |  | 76.63 | < .001 | .21 |  |  |  |
