# Supplementary material for: Urbanicity, biological stress system functioning and mental health in adolescents
Source: PLoS One. 2020 Mar 18;15(3):e0228659. doi: 10.1371/journal.pone.0228659 (PMC7080241; doi:10.1371/journal.pone.0228659)
Supplement: S4 Table — Bold = p < .01; italics = p < .05. BP = behavioral problems; EP = emotional problems; self and mother indicate the informant; AUCiHR = area under the curve with respect to ground, calculated for heart rate; MRHR = maximum heart rate response; AUCiC = area under the curve with respect to increase, calculated for cortisol; MRC = maximum cortisol response; AUCgC = area under the curve with respect to ground, calculated for cortisol; SESem = socioeconomic status, employment component; SESin = socioeconomic status, income component; SESfam = family socioeconomic status; Puberty = pubertal stage; BMI = body mass index; TDexercise = test day exercise; urbanicity, SES employment and SES income were measured at the neighborhood level. (DOCX) [file pone.0228659.s007.docx]

S4 Table

|  | 1 | 2 | 3 | 4 | 5 | 6 | 7 | 8 | 9 | 10 | 11 | 12 | 13 | 14 | 15 | 16 | 17 | 18 | 19 |
| --- | --- | --- | --- | --- | --- | --- | --- | --- | --- | --- | --- | --- | --- | --- | --- | --- | --- | --- | --- |
| 1.BP:self | - | **.40** | **.56** | **.26** | -.04 | **-.17** | -.07 | *-.14* | -.07 | -.02 | -.01 | -.06 | .03 | -.03 | -.00 | .02 | -.01 | .01 | .01 |
| 2.EP:self |  | - | **.21** | **.49** | .03 | .09 | .00 | -.06 | -.04 | .07 | -.08 | -.09 | -.04 | .05 | **.39** | *.13* | .09 | .03 | -.01 |
| 3.BP:mother |  |  | - | **.51** | .06 | **-.16** | **-.26** | -.10 | -.08 | -.00 | *-.12* | -.04 | -.07 | -.08 | .01 | -.11 | -.00 | .01 | -.06 |
| 4.EP:mother |  |  |  | - | .01 | -.02 | -.07 | -.11 | -.02 | .05 | -.10 | -.04 | -.08 | -.00 | **.25** | -.01 | .06 | .02 | -.08 |
| 5.Urbanicity |  |  |  |  | - | -.09 | **-.19** | .01 | -.06 | .02 | **-.60** | *-.12* | -.10 | .08 | .02 | -.06 | .08 | .06 | -.07 |
| 6.AUCiHR |  |  |  |  |  | - | **.37** | **.32** | **.19** | .09 | .05 | .05 | -.05 | .05 | *.16* | **.18** | -.04 | -.04 | .06 |
| 7.MRHR |  |  |  |  |  |  | - | **.23** | **.18** | *.13* | **.20** | .08 | **.21** | -.01 | .06 | .03 | *-.15* | .04 | -.01 |
| 8.AUCiC |  |  |  |  |  |  |  | - | **.36** | **.22** | .03 | *.12* | -.04 | *.13* | *-.12* | .07 | -.03 | *-.13* | .04 |
| 9.MRC |  |  |  |  |  |  |  |  | - | -.02 | .08 | .03 | .05 | **-.16** | -.10 | -.05 | -.03 | .06 | *-.12* |
| 10.AUCg |  |  |  |  |  |  |  |  |  | - | -.09 | -.04 | .04 | .03 | **.24** | .05 | .06 | -.03 | -.08 |
| 11.SESem |  |  |  |  |  |  |  |  |  |  | - | **.26** | *.11* | -.07 | -.08 | -.02 | **-.17** | .01 | .07 |
| 12.SESin |  |  |  |  |  |  |  |  |  |  |  | - | .11 | **.15** | -.10 | .07 | .01 | -.09 | *.13* |
| 13.SESfam |  |  |  |  |  |  |  |  |  |  |  |  | - | .03 | .06 | .04 | -.10 | .05 | .01 |
| 14.Age |  |  |  |  |  |  |  |  |  |  |  |  |  | - | -.03 | **.27** | *.13* | **-.15** | **.44** |
| 15.Sex |  |  |  |  |  |  |  |  |  |  |  |  |  |  | - | *.14* | *.14* | .00 | -.03 |
| 16.Puberty |  |  |  |  |  |  |  |  |  |  |  |  |  |  |  | - | **.19** | -.10 | **.21** |
| 17.BMI |  |  |  |  |  |  |  |  |  |  |  |  |  |  |  |  | - | -.00 | .09 |
| 18.TDexercise |  |  |  |  |  |  |  |  |  |  |  |  |  |  |  |  |  | - | -.08 |
| 19.Season |  |  |  |  |  |  |  |  |  |  |  |  |  |  |  |  |  |  | - |
