# Supplementary material for: Urbanicity, biological stress system functioning and mental health in adolescents
Source: PLoS One. 2020 Mar 18;15(3):e0228659. doi: 10.1371/journal.pone.0228659 (PMC7080241; doi:10.1371/journal.pone.0228659)
Supplement: S5 Table — Bold indicates p < .01; italics indicates p < .05; AUCiHR = area under the curve with respect to ground, calculated for heart rate; MRHR = maximum heart rate response; AUCiC = area under the curve with respect to increase, calculated for cortisol; MRC = maximum cortisol response; AUCgC = area under the curve with respect to ground, calculated for cortisol. Model fit indices were: CFI = .98, RMSEA = .03, SRMR = .04. (DOCX) [file pone.0228659.s008.docx]

S5 Table

|  | **Self-report** | | | | | **Mother-report** | | | | |
| --- | --- | --- | --- | --- | --- | --- | --- | --- | --- | --- |
|  | Est | SE | *z* | *p* | CI | Est | SE | *z* | *p* | CI |
| **Intercept** | -0.01 | 0.20 | -0.03 | .974 | -0.39/0.38 | -0.07 | 0.19 | -0.35 | .730 | -0.44/0.31 |
| **Indirect effects** | |  |  |  |  |  |  |  |  |  |
| AUCiHR | 0.01 | 0.01 | 1.18 | .237 | -0.01/0.03 | 0.00 | 0.01 | 0.08 | .937 | -0.01/0.01 |
| MRHR | 0.00 | 0.01 | 0.05 | .964 | -0.02/0.02 | *0.04* | *0.02* | *2.13* | *.033* | *0.00/0.07* |
| AUCiC | 0.00 | 0.01 | 0.25 | .806 | -0.01/0.02 | 0.00 | 0.01 | 0.24 | .807 | -0.01/0.01 |
| MRC | 0.00 | 0.01 | 0.43 | .667 | -0.02/0.02 | 0.00 | 0.01 | 0.21 | .832 | -0.02/0.02 |
| AUCg | -0.00 | 0.00 | -0.34 | .732 | -0.01/0.01 | -0.00 | 0.00 | -0.35 | .725 | -0.01/0.01 |
| **Direct effects** | |  |  |  |  |  |  |  |  |  |
| Urbanicity | -0.08 | 0.06 | -1.42 | .155 | -0.20/0.03 | 0.04 | 0.06 | 0.71 | .476 | -0.07/0.15 |
| AUCiHR | -0.13 | 0.07 | -1.82 | .069 | -0.27/0.01 | -0.01 | 0.07 | -0.08 | .937 | -0.14/0.13 |
| MRHR | -0.00 | 0.07 | -0.05 | .964 | -0.13/0.13 | **-0.23** | **0.07** | **-3.53** | **.000** | **-0.36/-0.10** |
| AUCiC | -0.12 | 0.07 | -1.68 | .092 | -0.27/0.02 | -0.10 | 0.07 | -1.40 | .163 | -0.24/0.04 |
| MRC | -0.03 | 0.07 | -0.44 | .663 | -0.16/0.10 | -0.01 | 0.07 | -0.21 | .832 | -0.14/0.11 |
| AUCg | 0.05 | 0.07 | 0.83 | .410 | -0.08/0.18 | 0.07 | 0.06 | 1.08 | .279 | -0.06/0.19 |
| Sex | -0.01 | 0.12 | -0.04 | .965 | -0.24/0.23 | 0.05 | 0.12 | 0.46 | .648 | -0.18/0.29 |
| Age | -0.01 | 0.06 | -0.11 | .915 | -0.13/0.12 | -0.03 | 0.06 | -0.53 | .599 | -0.14/0.08 |
