# Supplementary material for: Urbanicity, biological stress system functioning and mental health in adolescents
Source: PLoS One. 2020 Mar 18;15(3):e0228659. doi: 10.1371/journal.pone.0228659 (PMC7080241; doi:10.1371/journal.pone.0228659)
Supplement: S6 Table — Bold indicates p < .01; italics indicates p < .05; AUCi = area under the curve with respect to increase; MR = maximum response; Puberty = pubertal stage; BMI = body mass index; urbanicity was measured at the neighborhood level. Estimates are as reported in the model predicting behavioral problems, not controlling for socioeconomic status. (DOCX) [file pone.0228659.s009.docx]

S6 Table

|  | **Heart rate (AUCi)** | | | | | **Heart rate (MR)** | | | | |
| --- | --- | --- | --- | --- | --- | --- | --- | --- | --- | --- |
|  | Est | SE | *z* | *p* | CI | Est | SE | *z* | *p* | CI |
| **Intercept** | -0.75 | 0.39 | -1.94 | .053 | -1.50/0.01 | 0.01 | 0.06 | 0.12 | .903 | -0.11/0.12 |
| **Direct effects** | |  |  |  |  |  |  |  |  |  |
| Urbanicity | -0.09 | 0.06 | -1.58 | .113 | -0.21/0.02 | **-0.16** | **0.06** | **-2.68** | **.007** | **-0.27/-0.04** |
| Sex | 0.10 | 0.11 | 0.89 | .385 | -0.12/0.31 | - | - | - | - | - |
| Puberty | 0.14 | 0.08 | 1.68 | .093 | -0.02/0.31 | - | - | - | - | - |
| BMI | - | - | - | - | - | *-0.12* | *0.06* | *-2.14* | *.033* | *-0.23/-0.10* |
