# Supplementary material for: Urbanicity, biological stress system functioning and mental health in adolescents
Source: PLoS One. 2020 Mar 18;15(3):e0228659. doi: 10.1371/journal.pone.0228659 (PMC7080241; doi:10.1371/journal.pone.0228659)
Supplement: S7 Table — Bold indicates p < .01; italics indicates p < .05; AUCi = area under the curve with respect to increase; MR = maximum response; AUCg = area under the curve with respect to ground; TD exercise = test day exercise; urbanicity was measured at the neighborhood level. Estimates are as reported in the model predicting behavioral problems, not controlling for socioeconomic status. (DOCX) [file pone.0228659.s010.docx]

S7 Table

|  | **Cortisol (AUCi)** | | | | | **Cortisol (MR)** | | | | | **Cortisol (AUCg)** | | | | |
| --- | --- | --- | --- | --- | --- | --- | --- | --- | --- | --- | --- | --- | --- | --- | --- |
|  | Est | SE | *z* | *p* | CI | Est | SE | *z* | *p* | CI | Est | SE | *z* | *p* | CI |
| **Intercept** | **0.80** | **0.22** | **3.60** | **.000** | **0.36/1.23** | *0.38* | *0.17* | *2.20* | *.028* | *0.04/0.71* | **-0.70** | **0.19** | **-3.70** | **.000** | **-1.07/-0.33** |
| **Direct effects** | |  |  |  |  |  |  |  |  |  |  |  |  |  |  |
| Urbanicity | -0.01 | 0.06 | -0.25 | .804 | -0.12/0.10 | *-0.14* | *0.06* | *-2.44* | *.015* | *-0.26/-0.03* | -0.02 | 0.06 | -0.38 | .708 | -0.14/0.09 |
| Age | 0.03 | 0.05 | 0.60 | .549 | -0.07/0.13 | -0.09 | 0.06 | -1.40 | .163 | -0.20/0.03 | - | - | - | - | - |
| Sex | *-0.23* | *0.10* | *-2.30* | *.022* | *-0.42/-0.03* | - | - | - | - | - | **0.46** | **0.12** | **3.91** | **.000** | **0.23/0.70** |
| TD exercise | **-0.38** | **0.13** | **-3.01** | **.003** | **-0.63/-0.13** | - | - | - | - | - | - | - | - | - | - |
| Season | - | - | - | - | - | *-0.25* | *0.11* | *-2.25* | *.024* | *-0.46/-0.03* | - | - | - | - | - |
