# Supplementary material for: Urbanicity, biological stress system functioning and mental health in adolescents
Source: PLoS One. 2020 Mar 18;15(3):e0228659. doi: 10.1371/journal.pone.0228659 (PMC7080241; doi:10.1371/journal.pone.0228659)
Supplement: S8 Table — Bold indicates p < .01; italics indicates p < .05; AUCiHR = area under the curve with respect to ground, calculated for heart rate; MRHR = maximum heart rate response; AUCiC = area under the curve with respect to increase, calculated for cortisol; MRC = maximum cortisol response; AUCgC = area under the curve with respect to ground, calculated for cortisol. Model fit indices were: CFI = .97, RMSEA = .03, SRMR = .04. (DOCX) [file pone.0228659.s011.docx]

S8 Table

|  | **Self-report** | | | | | **Mother-report** | | | | |
| --- | --- | --- | --- | --- | --- | --- | --- | --- | --- | --- |
|  | Est | SE | *z* | *p* | CI | Est | SE | *z* | *p* | CI |
| **Intercept** | **-1.10** | **0.19** | **-5.91** | **.000** | **-1.46/-0.73** | **-0.69** | **0.19** | **-3.66** | **.000** | **-1.05/-0.32** |
| **Indirect effects** | |  |  |  |  |  |  |  |  |  |
| AUCiHR | -0.01 | 0.01 | -0.66 | .510 | -0.02/0.01 | -0.01 | 0.01 | -0.65 | .517 | -0.02/0.01 |
| MRHR | 0.01 | 0.01 | 0.46 | .643 | -0.02/0.03 | 0.03 | 0.01 | 1.79 | .073 | -0.00/0.05 |
| AUCiC | 0.00 | 0.01 | 0.30 | .762 | -0.01/0.02 | 0.00 | 0.01 | 0.31 | .760 | -0.02/0.02 |
| MRC | -0.01 | 0.01 | -0.77 | .444 | -0.03/0.01 | -0.01 | 0.01 | -0.98 | .328 | -0.03/0.01 |
| AUCg | -0.00 | 0.00 | -0.25 | .806 | -0.00/0.00 | -0.00 | 0.01 | -0.37 | .710 | -0.01/0.01 |
| **Direct effects** | |  |  |  |  |  |  |  |  |  |
| Urbanicity | -0.02 | 0.06 | -0.32 | .749 | -0.13/0.09 | 0.02 | 0.06 | 0.33 | .738 | -0.09/0.13 |
| AUCiHR | 0.05 | 0.07 | 0.73 | .468 | -0.09/0.19 | 0.05 | 0.07 | 0.71 | .478 | -0.09/0.18 |
| MRHR | -0.03 | 0.07 | -0.47 | .638 | -0.16/0.10 | *-0.16* | *0.07* | *-2.42* | *.016* | *-0.29/-0.03* |
| AUCiC | -0.11 | 0.07 | -1.57 | .117 | -0.25/0.03 | *-0.16* | *0.07* | *-2.35* | *.019* | *-0.30/-0.03* |
| MRC | 0.05 | 0.07 | 0.81 | .421 | -0.08/0.18 | 0.07 | 0.06 | 1.06 | .287 | -0.06/0.19 |
| AUCg | 0.02 | 0.06 | 0.30 | .763 | -0.10/0.14 | 0.09 | 0.07 | 1.32 | .187 | -0.04/0.22 |
| Sex | **0.70** | **0.12** | **6.02** | **.000** | **0.47/0.92** | **0.45** | **0.12** | **3.89** | **.000** | **0.23/0.68** |
| Age | 0.07 | 0.06 | 1.11 | .269 | -0.05/0.19 | 0.09 | 0.06 | 1.51 | 0.13 | -0.03/0.19 |
