# Supplementary material for: Urbanicity, biological stress system functioning and mental health in adolescents
Source: PLoS One. 2020 Mar 18;15(3):e0228659. doi: 10.1371/journal.pone.0228659 (PMC7080241; doi:10.1371/journal.pone.0228659)
Supplement: Attachment — Submitted filename: PONE-S-19-23797_Response to reviewers.docx [file pone.0228659.s012.docx]

We would like to thank the reviewers for their time and effort in reviewing our manuscript. In addition to the changes made in response to the comments below, we note that, in this revision, the model statistics are changed slightly due to updates to the lavaan package: <http://lavaan.ugent.be/history/dot6.html>. Specifically, this record states that: *the code to compute (robust) standard errors and (robust) test statistics has been rewritten; the code is now much more consistent, but this may result in small changes in the values of some robust test statistics as printed in the output of summary(); a meanstructure is no longer added automatically when switching to a robust estimator* (updated 22 may 2018). In addition, we reran the models using the full information maximum likelihood estimator which has since become available in the lavaan package.

**Reviewer #1**

Major Issues
1. Page 26 - lines 605 - 607 - Acknowledgements: This session includes funding sources, in discordance with the submission guidelines: “Funding information should only be entered in the financial disclosure section of the submission system1.”

*Response:* We took the funding information out of the Acknowledgements section.

2. Page 32 - lines 759-761 - References: Reference #56 references unavailable or unpublished work, according to PLOS ONE submission guidelines this data should be included as supplementary material or deposited in a publicly available database1. The guideline includes manuscripts that have been submitted but not yet accepted on this category1.

*Response:* This manuscript was submitted as supplementary information and we will do this again. The manuscript has since been conditionally accepted at BMC Psychology and is expected to be available soon.

3. Page 32 - lines 767-770 - References: References #59 and #60 are on the Open Science Framework, but are not readily available (available upon request only).

*Response:* These references have since been made public.

4. Page 12 - lines 312-313 - Measures: The content of content of the seven questions and the appearance of the visual thermometer is not revealed, which impairs the reproducibility of the experiment. Authors do inform methodology is pre-registered at the Open Science Framework, but it is not readily available.

*Response:* We included the Perceived Stress Questionnaire with a translation of the questions (S2 File) and the visual thermometer (S3 Figure) that were used. As noted above, the OSF registrations are now publically available.

Minor Issues

5. Page 2 - lines 30-31 - Abstract: It is not clear if what will be analyzed is the association in adolescents that grew up in urban areas or adolescents that are growing up in urban areas. It does become clear once we advance on the reading of the piece, but it would be interesting to clarify it from the start.

*Response:* We added ‘current’ to urbanicity in the abstract to clarify this (line 31).

6. Page 3 - lines 71-73 - Introduction: The passage makes reference to mental health in general, but the paper referenced (reference #14) is specifically on schizophrenia. It would be interesting to add some more references for this claim and I am sure the authors have plenty of literature collected on that.

*Response:* Thank you for this suggestion. We have revised this paragraph in light of some new work on selective migration. The corresponding reference in the revised version now refers to mental health in general. Please see lines 69-74.

7. Page 8 - line 216 - Measures: A brief explanation on the importance of cronbach's alpha would be advisable as is may deter readers not familiar with this measure. It would be interesting to consider the use of the term tau-equivalent reliability as there is some discussion about the interchangeability of the terms2. Also, it may be interesting to consider a congeneric reliability analysis.

*Response:* Thank you for bringing this to our attention. We changed the term to tau-equivalent reliability coefficient but kept Cronbach’s alpha in parentheses as this is still widely used, and included a brief explanation of it (lines 239-241).

8. Page 9 - lines 229-231 - Measures: The passage states the categorical coding of surrounding address density (SAD) was chosen to improve generalizability and that this is common practice in literature, but reference a single paper (that is not a review). It would be interesting to add some references to substantiate this claim.

*Response:* We included the references from two additional studies from the Netherlands that utilized this measure, as well as noted that this measure is recommended by Statistics Netherlands (line 257).

9. Page 9 - lines 232-234 - Measures: The passage introduces of biological stress system functioning utilized in the investigation (two indices of ANS reactivity: heart rate AUCi and MR; two indices of HPA axis reactivity: cortisol AUCi and MR; one index of basal HPA axis functioning: AUCgC). An early introduction of what these measures will be would improve the reading experience.

*Response:* We added a paragraph to the introduction explaining the different types of stress measures (lines 121-129) and took out some sentences in the discussion to avoid too much repetition.

10. Page 12 - lines 324-325 - Measures: It may be interesting to consider alternatives p-values considering recent discussions on the subject 3,4. It also would be interesting to add some comments about effect size and/or measures of association for the analysis that were statistically significant.

*Response:* We are aware of the discussions on the utility of p values and recognize the importance of providing alternatives to p values and of not mis-interpreting these statistics. We would like to stick to the pre-registered data plan in this study by maintaining our previously defined significance levels. We have added confidence intervals to the tables (please see Tables 2 through 5 and Supplementary Tables S9 through S12), and we removed the mention of marginal effects in the discussion. Also, we would like to note that we emphasize that most associations tested did not reach statistical significance.

**Reviewer #2**

Introduction:
11. Line 124: “adolescents seem to show different patterns of biological stress reactivity”. Could this be more specific? “different” could be anything. Regarding HR or cortisol levels, I assume there will be concrete evidence in one specific direction.

*Response:* Most evidence shows heightened HPA axis reactivity during adolescence, and ANS differences that depend on the specific measure. Heart rate recovery becomes quicker during adolescence (Gunnar, Wewerka, Frenn, Long, & Griggs, 2009). We revised the sentence so that it is more specific (line 135).

12. Lines 130-133: “Results from two neuroimaging studies“ is followed by 3 references. This does not match

*Response:* The Lederbogen, et al (2011) and Streit, et al (2014) are independent studies, whereas the Lederbogen, et al (2013) is a review. We took out the ‘two’ in the sentence to avoid readers perceiving a mismatch as well.

13. Lines 143-147: the reference to the lines feels counter-intuitive. I would suggest to call them A, B and C in order, A resulting in the direct association, and B and C as parts of the indirect way.

*Response:* We changed the figure so that the A and B are the top arrows in the figure, and C below. We would prefer to keep our notation of the model in line with the common notation of the mediation model, in which C is the direct association (e.g. (Baron & Kenny, 1986; MacKinnon & Fairchild, 2009).

14. Lines 149-150: Here the authors state that the hypothesis was rejected in children. However, the motivation and rationale to investigate the same hypothesis in adolescents may be elaborated on. Why would the factors cited, contributing to vulnerability to mental health problems (lines 152-153) make it plausible that this hypothesis might be true for adolescents? Additionally, comparing it more to the adult literature in light of this specific hypothesis would also validate this choice of hypothesis more.

*Response:* Thank you for this suggestion. We elaborated on this hypothesis and included a comparison to the findings on urbanicity and mental health in youth compared to adults. Please see lines 162-169.

15. Line 158, Note to figure 1: the factors mental health and biological stress functioning are well described. Pleas add a definition of urbanicity here as well, to complete the information.

*Response:* We included a definition of urbanicity in Figure 1 (lines 176-177).

Materials and Methods

16. Lines 197-198: explaining the procedure of saliva collection, a reference to Figure 2 would be helpful.

*Response:* We have added a reference to Figure 2 as suggested (now line 214).

17. Urbanicity: Please state here that the measure investigated is current urbanicity. Further in the discussion this is done, but here should be the place.
To my knowledge, all Dutch studies using CBS data on SAD use a 5 scale division of urbanicity (see for example Van Os, 2002, Frissen 2014/2015 and Peeters 2015). This is also the division given on the CBS website. (<https://urlproxy.sunet.se/canit/urlproxy.php?_q=aHR0cHM6Ly9qZXVnZG1vbml0b3IuY2JzLm5sL2JlZ3JpcHBlbi1lbi10b2VsaWNodGluZ2VuL3N0ZWRlbGlqa2hlaWQtdmFuLWVlbi1nZWJpZWQ%3D&_s=YnJpdHRhbnkuZXZhbnNAa2F1LnNl&_c=6d3817ab&_r=a2F1LXNl>): “Hierbij zijn vijf categorieën onderscheiden: - zeer sterk stedelijk: gemiddelde oad van 2500 of meer adressen per km2; - sterk stedelijk: gemiddelde oad van 1500 tot 2500 adressen per km2; - matig stedelijk: gemiddelde oad van 1000 tot 1500 adressen per km2; - weinig stedelijk: gemiddelde oad van 500 tot 1000 adressen per km2; - niet stedelijk: gemiddelde oad van minder dan 500 adressen per km2.”
The 5 scale division should be adopted in the manuscript.
Furthermore, in Table 1 urbanicity is given as a mean, but the n per category is much more informative. Were participants equally distributed over the categories, or were they predominantly urban? Since urbanicity is a very important component in this manuscript, it warrants more detailed description and should be comparable to other Dutch sample studies.
And to conclude, in the discussion (lines 533-540) studies of urban upbringing and current city living are compared, as if these measured the same construct. Possibly the different definitions and operationalizations of urbanicity may account for differences in outcome as well.

*Response:* We changed ‘urbanicity’ to ‘current urbanicity’ in the method section (please see lines 242-243, 250). We did indeed use the 5-category division of urbanicity as suggested by Statistics Netherlands (please see the description on lines 250-255). We added an additional mention of this at the beginning of the same paragraph (line 250) in order to clarify this. We also added the percentages of individuals in each of the 5 categories of the urbanicity scale in S8 Figure, referred to in the text on lines 393-394. We added a mention of the inconsistency between current urbanicity and urban upbringing as suggested in the discussion (now line 549-550).

18. Lines 267-269: by choosing the extremes of the measurement points (RC1 and 2; RC 3, 4 and 5) it feels like blowing up the differences. I strongly believe that an average would be a more valid value. Please adjust this.

*Response:* In measuring the biological stress response, we are interested in the reactivity of the system, in other words, we are interested in the difference between stress system output during rest and during stress. There is a wide variation in individuals’ stress responsivity, therefore, for some the first measurement may be the best reflection of a resting state, whereas others may begin the session with somewhat higher stress levels due to anticipation of the upcoming tasks, and therefore the second measurement may be a better reflection of the resting state. Similarly, some individuals respond physiologically more strongly to some tasks (e.g. serial subtraction) whereas others respond more strongly to other tasks (e.g. public speaking). We are more interested in the response itself, and not to differences between tasks. The minimum-maximum calculation as an index of acute stress reactivity is therefore often used in this kind of research (e.g. (Alink et al., 2008; Kirschbaum, Pirke, & Hellhammer, 1993; Linden, Earle, Gerin, & Christenfeld, 1997; Miller et al., 2018). We have added references to the method section (lines 275 and 289).

19. Line 286: T2 n = 1105. But in the participants section, the authors state that T2 data were used (N = 990). And in line 316 509 adolescents participated in the T2 measurement. Somehow these different numbers are confusing and it is not clear on what these differences are based. Please clarify.

*Response:* In order to clarify this, we added a flow chart of the data inclusion procedure and available data (S1 Figure). We refer to this in the manuscript text on lines 195 and 343.

20. Line 324: the participating sample differed from the whole sample in fewer problems, lower neighborhood SES, and older age. The way I read the Table S1 I see MORE self-reported behavioral problems, HIGHER neighborhood SES and older age.

*Response:* Your interpretation is correct. Thank you for noticing this mistake. It has been corrected (line 345).

21. In Lines 297 and further covariates are described. In line 349 it is explained that the analyses are controlled for these variables, but only in the preliminary results it becomes clear which covariate is used in which analysis. Maybe I have missed it, but what is the basis for this decision, and could the specific use be reported earlier?

*Response:* We included our method for determining which covariates to include in the analyses (we examined bivariate correlations between the potential covariates and the stress indices) in the measures section so that it is clear earlier on (lines 326-328).

Discussion:
22. Lines 477-478: urbanicity is associated with mental health problems in adolescence. I would suggest to be more specific here: only in the behavioral outcome measures such an association was found, and not in the emotional problems. “problems in mental health” therefore seems too strong.

*Response:* We agree and have changed ‘mental health’ to ‘behavioral problems’ (lines 483 and 486).

23. Lines 514-515: urbanicity is associated with acute measures, but not with overall. This is a finding that needs explanation. Is there a specific mechanism that could underlie this relation with acute but not general stress reactivity? I would very much like a bit more discussion than the mere statement about this interesting finding.

*Response:* We agree that this is an interesting finding, and discuss it a bit further in two paragraphs following the noted sentence. We added more discussion of this in the suggested place as well (lines 523-529).

24. Line 584: comorbidity might be too strong a word for a healthy sample with merely subclinical symptoms.

*Response:* We have replaced ‘comorbidity’ with ‘association between behavioral and emotional problems’ (lines 598-599).

25. Line 196: Electrocardio-activity (without a space)

*Response:* This has been corrected (line 214).

26. Line 364: “Table” should be removed

*Response:* This has been corrected.

**Reviewer #3**

27. 1. The theoretical part and introdcution summarize findings on stress effects on mental health, effects of urbanicity on mental health, and associations with stress biomarkers. The mediation model tested in this ms., however, seems poorly prepared on a conceptual level - why is it that the authors think urbanicity effects on metal health are mediated by stress biomarkers? Why would stress markers not just be a correlate of urbanicity? This needs to be explicated in more detail.

*Response:* We elaborated on why we hypothesized that stress system functioning might explain the association between urbanicity and mental health at the end of the introduction, please see lines 162-170, as well as our response to comment 14.

28. 2. Please clarify the use of zip codes. How large is the variation of the population size in a neighbourhood (mean 1400) in the Netherlands? How was the 1km radius implemented? By address or by center of zip code?

*Response:* The range of population size of neighborhoods can vary from 35 to 28,380 (as of 2018). We added this information to the manuscript (lines 245-246). The 1 km radius is implemented by address: the surrounding address density is the number of addresses within a circle with a 1 km radius around an address, divided by the area of the circle. This is noted in the manuscript (line 252-253).

29. 3. One theoretical model of urbanicity implies that population density is inversely associated with socioeconomic status. In your mediation analyses, when controlling for SES, the problem arises that you may have controlled for a key mechanism and hence lost the effect of interest. Please also show analyses including SES as a mediator or at least not controlling for SES.

*Response:* We agree that controlling for SES may effectively mean controlling for a major mechanism of the urbanicity-mental health association. Indeed, urbanicity and neighborhood SES were inversely correlated in our study (S7 Table). However, our aim in this study was to examine the effects of urbanicity independent of socioeconomic conditions, in order to more closely examine the associations with biological stress system functioning. We did run our analyses again without controlling for neighborhood and family SES and report this as a supplementary analysis (lines 371-373). The results are reported in Supplementary Tables S9 through S12 and in the text (lines 412-413, 422-423 and 582-586).

30. 4. Given the high number of correlations under investigation as well as the preselection of variables based on significant associations, how did you control for multiple testing?
*Response:* We took a modelling approach to answer this research question in order to reduce the number of tests involved. Given that we had multiple informants, and considering the correlations between the constructs, we felt that a structural equation model would be the most appropriate method. Thus, all constructs are modelled simultaneously. We did not apply additional controls for multiple testing. Bonferonni correction is considered to be too conservative for structural equation models.

31. 5. Please demonstrate how your analyses are powered (e.g. post hoc) for the mediation analyses provided here.

*Response:* We performed a post hoc power analysis and report the results of this in the data analysis section (please see lines 379-383).

Alink, L. R. A., van Ijzendoorn, M. H., Bakermans-Kranenburg, M. J., Mesman, J., Juffer, F., & Koot, H. M. (2008). Cortisol and externalizing behavior in children and adolescents: Mixed meta-analytic evidence for the inverse relation of basal cortisol and cortisol reactivity with externalizing behavior. *Developmental Psychobiology, 50*(5), 427-450. doi:10.1002/dev.20300

Baron, R. M., & Kenny, D. A. (1986). The moderator mediator variable distinction in social psychological research: Conceptual, strategic and statistical considerations. *Journal of Personality and Social Psychology, 51*(6), 1173-1182. doi:10.1037//0022-3514.51.6.1173

Gunnar, M. R., Wewerka, S., Frenn, K., Long, J. D., & Griggs, C. (2009). Developmental changes in hypothalamus-pituitary-adrenal activity over the transition to adolescence: Normative changes and associations with puberty. *Development and Psychopathology, 21*(1), 69-85.

Kirschbaum, C., Pirke, K.-M., & Hellhammer, D. H. (1993). The 'Trier Social Stress Test': A tool for investigating psychobiological stress responses in a laboratory setting. *Neuropsychobiology, 28*(1-2), 76-81.

Linden, W., Earle, T. L., Gerin, W., & Christenfeld, N. (1997). Physiological stress reactivity and recovery: Conceptual siblings separated at birth? *Journal of Psychosomatic Research, 42*(2), 117-135. doi:10.1016/s0022-3999(96)00240-1

MacKinnon, D. P., & Fairchild, A. J. (2009). Current directions in mediation analysis. *Current Directions in Psychological Science, 18*(1), 16-20. doi:10.1111/j.1467-8721.2009.01598.x

Miller, R., Wojtyniak, J. G., Weckesser, L. J., Alexander, N. C., Engert, V., & Lehr, T. (2018). How to disentangle psychobiological stress reactivity and recovery: A for comparison of model-based and non-compartmental analyses of cortisol concentrations. *Psychoneuroendocrinology, 90*, 194-210. doi:10.1016/j.psyneuen.2017.12.019
